# Primary Hydatid Cyst: An Unusual Cause of a Mass in the Supraclavicular Region of the Neck

**DOI:** 10.4021/jocmr495w

**Published:** 2011-02-12

**Authors:** Ismail Iynen, Ozgur Sogut, Muhammet E Guldur, Rustu Kose, Halil Kaya, Ferhat Bozkus

**Affiliations:** a Department of Head and Neck Surgery, Harran University, Faculty of Medicine, Sanliurfa, Turkey; b Department of Emergency Medicine, Harran University, Faculty of Medicine, Sanliurfa, Turkey; c Department of Pathology, Harran University, Faculty of Medicine, Sanliurfa, Turkey; d Department of Plastic and Reconstructive Surgery, Rize University, Faculty of Medicine, Rize, Turkey

## Abstract

Hydatid disease, also known as echinococcosis or hydatidosis, is an infectious disease caused by the cestode Echinococcus. Echinococcus granulosus is the most common Echinococcus species affecting human beings. It may affect any organ and tissue in the body, in particular the liver and lung. Musculoskeletal or soft tissue hydatidosis accounts for about 0.5% 5% of all echinococcal infections in endemic areas, and is almost always secondary to the hepatic or pulmonary disease. Even in regions where echinococcosis is endemic, hydatidosis of cervicofacial region is extremely rare. Herein, we present exceptionally rare case with an unusual localization of primary hydatid cyst in the left supraclavicular region of the neck.

**Keywords:**

Hydatid cyst; Supraclavicular region; Neck; Unusual localization

## Introduction

Hydatid disease, also known as echinococcosis or hydatidosis, is a zoonotic infection caused by the larval forms (metacestode) of *Echinococcus granulosus* that lives in the small intestines of adult dogs [1]. Hydatic cyst is a prevalent parasitic disease in the Middle east, Mediterranean countries, South America, North Africa and Australia. In humans the hydatid disease commonly involves the liver (70%) and the lungs (25%) [2]. The spleen, kidneys, bile ducts, mesentery, heart, brain and muscluloskeletal or soft tissue are less frequent sites of the involvement in hydatid disease [2, 3]. Its symptoms may emerge depending on the host organ, location, its effect on adjacent structures, complications due to rupture, secondary infections, and immunological reactions caused by the cyst [2]. Definite diagnosis is mostly based on cross-sectional imaging techniques such as ultrasound (US), computed tomography (CT) or magnetic resonance imaging (MRI) [3].

Even in regions where echinococcosis is endemic, hydatidosis of neck is rare and its incidence is unknown. In this paper, a case with an unusual localization of primary hydatid cyst in the left supraclavicular region of the neck is presented and available diagnostic tests and therapeutic approaches are discussed in the view of literature.

## Case Report

**Figure 1.**
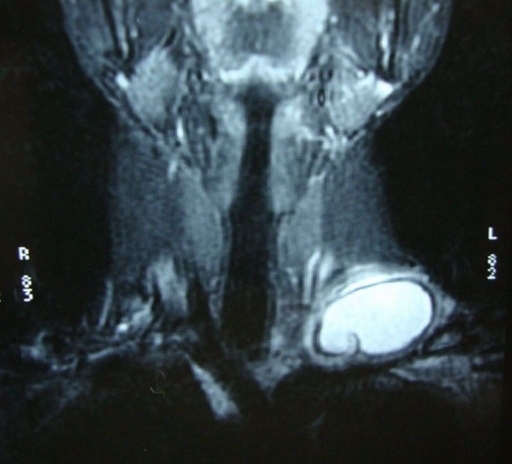
Coronal T2-weighted MRI showing a unilocular, well-defined and high signal intensity cystic lesion with hypointense rim.

**Figure 2.**
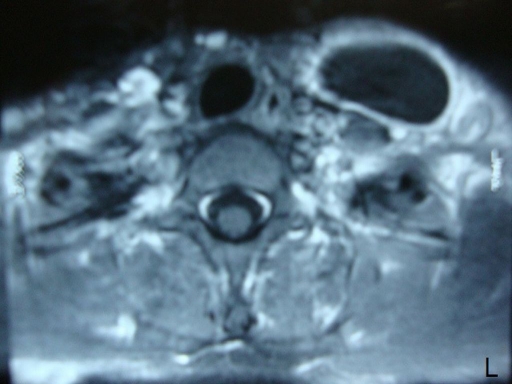
Contrast-enhanced axial T1-weighted MRI showing a well-defined hypointense cystic mass with a periferic enhancement.

**Figure 3.**
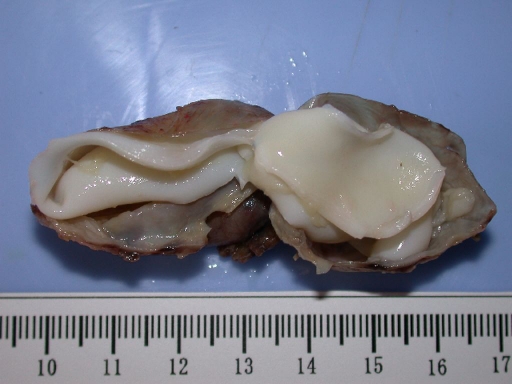
A view of the resected cyst and its germinative membrane.

**Figure 4.**
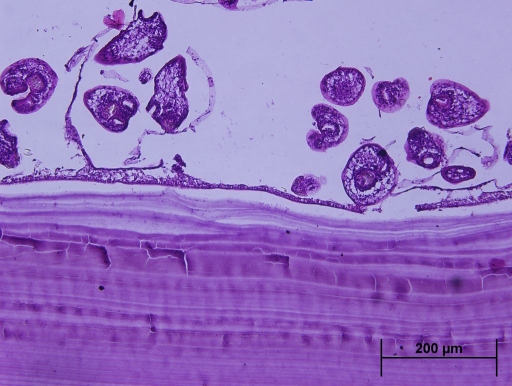
Microscopic examination of the specimen demonstrating a thick collagenous fibrotic cyst wall surrounded by a striated muscle fibril and a free scolex in the cyst lumen.

A 21-year-old woman presented to our hospital with a three month history of painless lump growing on the left lateral side of her neck. Upon physical examination, there was a uniform mobile, smoothly outlined mass with a diameter of 5 x 4 cm on the supraclavicular region of the left lateral neck. The mass did not reveal any findings of fluctuation, erythema, ecchymosis or regional lymphadenopathy. Ultrasonographic examination of the mass showed a homogenous and anechoic cyst with a slightly thick wall measuring 4 x 5 x 5 cm in the left supraclavicular region. Lateral X-ray of neck and cervical spine revealed a mass without bone involvement. A coronal T2-weighted magnetic resonance imaging (MRI) of the left anterior neck showed a unilocular, well-defined and high signal intensity cystic lesion with hypointense rim, which is a characteristic feature for hydatid cyst (Fig. 1). A contrast-enhanced axial T1-weighted MRI showed a well-defined hypointense mass with periferic enhancement (Fig. 2). Abdominal and chest X-rays and ultrasound revealed no evidence of hepatic, pulmonary or other involvements. We suspected primary hydatid disease of the left neck, as this is an endemic region. Hemagglutination test of the patient for hydatid disease was positive. Complete surgical resection of the cystic mass from the surrounding muscle tissue was performed without rupture. Histopathological examination of the specimen confirmed the diagnosis of Echinococcus cyst. The gross specimen comprised of soft, smooth-walled and encysted structures measuring 4 cm in diameter. Unilocular cyst with clear fluid and membranous structures inside was observed in the sections of the specimen (Fig. 3). Microscopic examination showed the typical fibrolamellar membrane and several scolexes on the cyst wall (Fig. 4). The patient was discharged from the hospital with a prescription of albendazole 400 mg twice a day for four weeks.

## Discussion

Hydatid cyst is an infectious disease which is most commonly caused by the cestode parasite (tapeworm), Echinococcus granulosus and less commonly by echinococcus multilocularis [4]. Dogs are the main host, and animals like cattle, sheep, horse and pig are intermediate hosts in the disease. Parasite eggs that penetrate the organism hatch in the small intestine of the main host, pass into portal venous system or lymphatic system and reach the liver and lungs, and finally form hydatid cyst lesions. Moreover, they can cross the hepatic sinusoid or pulmonary capillary barriers, and embryos get into systemic circulation and can settle in all the organs and structures in the body [1, 4]. Hydatid cyst is most frequently involved in the liver and lungs, and rarely in the bone, brain, eye, heart, kidney and spleen [3]. It is extremely rare in the cervicofacial region, and its incidence is unknown [5]. Atypical localization of hydatid cyst may be challenged the diagnosis of hydatid disease [2]. Chevalier et al have reported the subcutaneous cyst hydatid incidence as 2%. However, this ratio also includes the secondary hydatid cyst cases [6]. Although the disease is generally asymptomatic, it may exhibit clinical symptoms depending on the size and location of the cyst, and the pressure of the growing cyst [7]. In our patient, there were no symptoms except a painless and mobile lump in the left supraclavicular area.

Echinococcosis is diagnosed essentially by the patient's history, physical examination findings, radiologic imaging modalities, aspiration and serological tests. Radiologic imaging modalities generally include ultrasound, computerized tomography (CT) and MRI [6]. Serological tests are indirect hemagglutination, latex agglutination, ELISA and immunoelectrophoresis. In addition, false positive and false negative results can be obtained by these tests [7]. We performed hemagglutination test in our patient and the result was positive. Abdominal and chest X-rays, ultrasound and CT scans should be performed in order to investigate various organ involvements, particularly liver and lungs [3]. Eroglu et al reported an unusual case of hydatid cyst found in the neck of a 66-year-old Turkish woman, and just like our case report, there was no pulmonary or hepatic involvement [8].

In the period from the first infection to clinical symptoms, echinococcosis displays the characteristic symptoms of benign tumor that grows slowly. Clinical symptoms depend on the anatomic host area. It may imitate benign and malign tumors, cysts, abscess, hematoma, pseudocyst and congenital cysts [7, 9].

Cystectomy (surgical excision) is the single effective treatment in hydatid cyst treatment and sufficient by itself. The prognosis is excellent in hydatid cyst cases who are treated by removal of cyst totally without rupture [3, 10, 11]. In order to prevent recurrence, the cyst should be protected against rupture during operation. Albendazol can be used postoperatively and preoperatively for the purpose of cyst size reduction [10, 11]. Total excision of the cystic mass was performed surgically in our patient. We continually followed our case during 1-year period, and we have not observed recurrence yet. Avcu et al [12] reported an unusual case with a surgical excision of hydatid cyst in submandibular and thyroid gland instead of holding PAIR (puncture-aspiration of cyst contents-injection of hypertonic saline solution-reaspiration) practiced with success.

In conclusion, hydatid disease is a widespread public health problem in developing countries. The possibility of hydatid disease, especially in endemic regions, should always be considered in the differential diagnosis of mesencyhmal neoplasms or soft tissue masses in the neck or in the other parts of the body. Radiologic imaging modalities in such cases are mandatory for the diagnosis of unilocular or multilocular hydatid cyst with thin borders, thin walls, inner membranes, and a distinct appearance of characteristic cystic mass. The prognosis is excellent in hydatid cyst cases treated with total removal of the cyst without rupture.
